# Invasive fungal infections in children with leukemia in a tertiary hospital in Oman: An eight-year review

**DOI:** 10.22034/CMM.2023.345108.1447

**Published:** 2023-09

**Authors:** Hind Al Hajri, Widad Al-Salmi, Karima Al Hinai, Saif Al-Housni, Ahmed Al-Harrasi, Hilal Al Hashami, Abdullah M.S. Al-Hatmi

**Affiliations:** 1 Oman Medical Specialty Board, Muscat, Oman; 2 Department of Paediatric Haematology and Oncology, Royal Hospital, Muscat, Oman; 3 Natural and Medical Sciences Research Centre, University of Nizwa, Nizwa, Oman; 4 Department of Paediatrics Infectious Disease, Royal Hospital, Muscat, Oman; 5 Center of Expertise in Mycology, Radboud University Medical Center/Canisius Wilhelmina Hospital, Nijmegen, The Netherlands

**Keywords:** Acute lymphoblastic leukemia, Antifungal, *Aspergillus*, *Candida*, Children, Invasive fungal infection, Prevalence

## Abstract

**Background and Purpose:**

Invasive fungal disease (IFD) is a common and serious consequence of leukemia in children and the incidence of these infections has increased due to chemotherapy. This study aimed to present the epidemiology of IFD in a cohort of children with leukemia from a tertiary reference institution in Oman.

**Materials and Methods:**

A retrospective study of IFDs in pediatric patients below 13 years of age with newly diagnosed or relapsed leukemia was conducted at the Royal Hospital in Muscat, Oman. From 2010 to 2017, IFD episodes in children with leukemia were evaluated retrospectively, considering age, gender, type of leukemia, chemotherapy regimen, IFD detection phase, neutropenia, prevention, diagnostic method, and treatment.

**Results:**

Between 2010 and 2017, 198 children with leukemia were admitted and treated at Royal Hospital. Invasive fungal infection (IFI) was diagnosed in 32 patients out of 198 (16.1%), and IFI was defined as probable and proven in 53% (n=17) and 47% (n=15) of the cases, respectively. At 1.1:1, the male-to-female ratio was roughly equal. According to chest computed tomography scans, 65.6% of patients had radiological features of fungal infections. Positive fungal cultures were found in the bronchoalveolar lavage of three patients, 37.5% of whom had positive blood cultures, and 3% had positive urine cultures as a neonatal invasive candidiasis. In three patients, invasive aspergillosis caused pulmonary IFD, accounting for 9.3% of all infection sites. Candidaemia was found in 28% of IFD patients, and the most common organism was *Candida tropicalis* (15.6%), followed by *Candida parapsilosis* (6.25%). Furthermore, the major risk factor was febrile neutropenia.

**Conclusion:**

In children with leukemia, invasive fungal infection is common and serious. Despite aggressive treatment, mortality among these high-risk patients remains high.

## Introduction

Invasive fungal infection (IFI) is a prominent cause of morbidity and mortality in immunocompromised people, especially children with cancer and those undergoing hematopoietic stem cell transplantation (HSCT) [ 1 , 2 ]. Majority of IFIs have an impact on oncology patients with chronic neutropenia [ 2 , 3 ]. Patients with acute myeloid leukemia (AML), recurrent acute leukemia, high-risk acute lymphoblastic leukemia, and allogeneic HSCT are more likely to develop IFI [ 4 ].

The IFI in cancer-stricken children continues to be a problem due to the lack of sensitivity and specificity of available diagnostic tools, or since they take too long to yield a clinically significant result [ 5 ]. Diagnoses of IFI in cancer patients and HSCT recipients have been classified as proven, probable, or possible by the European Organization for Research and Treatment of Cancer/Invasive Fungal Infections Cooperative Group and the National Institute of Allergy and Infectious Diseases Mycoses Study Group (EORTC/MSG) [ 6 ]. Despite advancements in non-culture-based technologies, such as β-glucan, galactomannan assays, lateral-flow devices, polymerase chain reaction, and antifungal susceptibility testing, the detection, and treatment of IFI promptly is still a challenge [ 7 ].

Many studies have investigated the features of IFIs in patients with hematological malignancies, but only a few have investigated the children population. Leukaemia is the most common malignancy in children under the age of 15, accounting for more than a third of all cancers in this age group [ 8 ]. Development of antifungal strategies to treat and prevent IFI in high-risk groups requires a thorough understanding of local IFI epidemiology. According to Al Lamki et al., the incidence rate of cancer among children under the age of 12 years in Oman between 1988 and 1992 was 11.2% [ 9 ]. Furthermore, according to a regional study conducted over four years from 1998 to 2001, the incidence rate of malignancy in the pediatric population under 15 years of age in Oman was 9.2 % [ 10 ]. Al-Hatmi et al. in 2021 assessed the burden of fungal infections in Oman and reported that the crude incidence of all malignancies in males was 63.9/100,000 in 2018, with a total of 2,940 cases. Leukemia was found to be present in 7.6% of the population (n=223), with AML accounting for 30% of them (n=67). Females had a crude cancer rate of 74.9 per 100,000 (total of 3447), with a 4.73% leukemia prevalence [ 11 ]. 

Data on hematological disorders were provided by cancer incidence in Oman in 2015 (Department of Non‐Communicable Disease) [ 12 ]. In Oman, two patients with thalassemia were diagnosed with invasive aspergillosis (IA) and successfully treated with aggressive systemic antifungal therapy for severe invasive pulmonary *Aspergillus* [ 13 ]. Invasive aspergillosis was also represented in hematological illnesses as AML and non-AML disorders, which comprised all lymphomas, non-AML leukemia, multiple myeloma, and myelodysplastic syndromes [ 11 ]. Although both children and adults are vulnerable to IFI, there are important differences to consider between these two groups of patients. 

Management options are influenced by epide-miology, diagnostic procedures, antifungal agent selection, and results. Over time, the overall survival rate of pediatric acute leukemia has increased, reaching 90% for acute lymphoblastic leukemia (ALL) and 70% for AML [ 14 ]. According to our knowledge, no research has been conducted in Oman on fungal infections in leukemic patients. As a result, the current study aimed to investigate the clinical spectrum, epidemiology, and outcomes of IFI in leukemic pediatric patients at a tertiary medical center in Muscat, Oman.

## Materials and Methods

### Samples and setting

Between 2010 and 2017, the medical records of pediatric patients with acute leukemia (below 13 years old) diagnosed at the Paediatric Haematology and Oncology Departments of Royal Hospital were reviewed individually and thoroughly. The Royal Hospital is a referral center for children diagnosed with hematologic malignancies, with a 24-bed capacity and pediatric intensive care unit (PICU) backup. This evaluation focused on children with leukemia who were treated at the reference oncology center in Oman. In this study, the state of leukemia, chemotherapy protocol and agent, and duration and severity of neutropenia (if any) in each course of chemotherapy were recorded. 

Data were collected using an electronic healthcare system after receiving ethical approval from the Research Committee of the center. This study included only children with ALL and AML, excluding oncology patients with solid organ cancers, hematopoietic stem cell recipients, and patients undergoing a bone marrow transplant (BMT). Moreover, there were no post-BMT patients included in the study. The Ethics Committee at Royal Hospital, Ministry of Health (SRC #101/2017) approved the present study.

### Data collection

During the research period, 198 patients were diagnosed with leukemia and their data were evaluated. The febrile neutropenia attacks were studied to define and classify IFI using the EORTC/MSG Consensus Group [ 15 ]. According to this classification, the presence of a host factor, a clinical criterion, and a mycological criterion is required for probable IFI. Cases that meet the criteria for a host factor and the clinical criterion but do not meet the criteria for a mycological factor are considered possible IFI. 

In the microbiology laboratory, traditional culture, microscopic examination, and histopathology were used to recover fungal isolates, as well as Matrix-assisted laser desorption/ionization-time of flight, MALDI-TOF (Bruker Daltonics^TM^) for final identification of these isolates. Any fungus detected by histological analysis or culture of a tissue specimen taken from a disease site is referred to as a proven IFI. Files of patients were examined for any microbiological and/or radiological evidence or signs of fungal infection. Regarding those who had evidence of fungal infection, demographic information, such as age and gender, type of leukemia, chemotherapy regimen, phase of IFI discovery, neutropenia, and thrombocytopenia status, use of prophylactic antifungal agents, diagnostic method, type of antifungal drug, treatment duration, and outcome were extracted from their files. 

### Statistical analysis

Data was first collected using an Excel application, and then all statistical analyses were performed in SPSS software (version 23.0). Means and medians were used to present descriptive data. The correlation between different factors was evaluated using descriptive analysis.

### Ethical Considerations

This study was approved by the Ethics Committee of the Royal Hospital, Ministry of Health, Oman (SRC #101/2017) 

## Results

### Prevalence and risk factors

**Figure 1:**
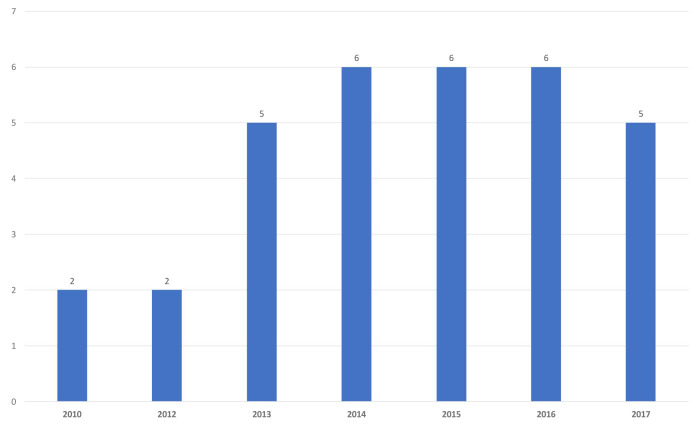
Number of fungal infections per year

Thirty-two episodes of IFI were included in the analysis over 8 years (Figure 1). These cases were either proven or probable, according to the European Organisation for Research and Treatment of Cancer criteria, yielding a rate of 16.1% (32/198). The children in this cohort were within the age range of 4 months to 13 years old, with an average of 4.9 years of age. Males and females were both affected in roughly equal proportions. The ALL was the most common subtype, accounting for 57% of all patients, followed by AML, which accounted for 15% of them. Relapsed ALL, relapsed AML, infantile leukemia, and mixed ALL/AML phenotype leukemia accounted for 11%, 3%, 7%, and 3% of total patients, respectively (Table 1). 

**Table 1:** Characteristics of 32 children with acute lymphoblastic leukemia and acute myeloid leukemia who had fungal infections between 2010 and 2017

Characteristic	No. of patients
Total	32
Age range (year)	4 months-13 years
Gender (male: female)	18 (56.3%): 14 (43.8%)
Types of leukaemia	
All	22 (68.75%)
AML	4 (12.5%)
Mixed phenotype	1 (3.13%)
Infantile ALL	2 (6.25%)
Relapsed ALL	2 (6.25%))
Relapsed AML	1 (3.13%)
Phase of treatment	
Induction	14 (43.8%)
Consolidation	3 (9.4%)
Intensification	4 (12.5%)
Maintenance	9 (28.1%)
FLAG-Ida	2 (6%)
Mycological test (direct/indirect)	
Blood culture	(37.5%)
FNA/wound culture	(18%)
Urine culture	(3%)
Radiology	(65.6%)
Diagnosis on admission	
FN	(62%)
Pneumonia	(18%)
Septic shock	(3%)
Treatment	
Liposomal amphotericin B	21
Voriconazole	32
Fluconazole	1
Posaconazole	1
Caspofungin	8
ICU admission	
Admitted	(56%)
Not admitted	(44%)
Outcome	
Survived	81%
Died	19%

ALL: acute lymphoblastic leukemia, AML: acute myeloid leukemia, FLAG-Ida: fludarabine, cytarabine, granulocyte-colony stimulating factor, and idarubicin, FNA: fine needle aspiration, FN: febrile neutropenia

The median number of days before starting antifungal medication as treatment was four, and the median number of days for each IFI episode and neutropenia episode was 19. The vast majority of our patients had thrombocytopenia. The initial diagnosis in 20 patients was febrile neutropenia (62.5%). Six patients (18.75%) were diagnosed with pneumonia with chest X-ray consolidation and later confirmed by positive CT chest for fungal infection, and six more (18.75%) were admitted with a septic shock-like picture. However, all of the patients eventually developed febrile neutropenia. During the infection episodes, the majority of patients (84%) (27/32) had a central line. Furthermore, in this study, the majority of the 32 identified IFI cases were candidemia, followed by invasive aspergillosis and mucormycosis.

### Microbiology and radiology investigation

Furthermore, radiological imaging, such as a CT scan of the chest, revealed evidence of fungal infection (in the form of ground glass appearance, nodular shadow, and air crescent signs) in 21 patients (65.6%). It should be mentioned that bronchoalveolar lavage (BAL) fungal culture was positive and negative in three and four of them. However, antifungal therapy had already begun empirically before CT. The remaining 11 children had not been given BAL samples since their families refused or they were already responding well to empirical antifungal treatment. 

*Aspergillus* pleural fluid was positive in one child with positive chest CT findings for fungal infection with complicated pleural effusion that required chest drain. However, a BAL sample was not performed as the child was very sick and admitted to the PICU. In BAL cultures, samples of three patients were positive for *Aspergillus fumigatus*, and one tested positive for *Aspergillus flavus*. Overall, Aspergillus was found in the pleural fluid cytology sample of one patient by direct visualization of characteristic hyphae while four patients had pulmonary aspergillosis, and 17 others had lung fungal infections. 

Furthermore, a child with *Candida tropicalis* positive blood culture had several abscesses as well as a gluteal abscess shown on abdominal CT. Another child tested positive for *Candida tropicalis* with several micro-abscesses in the lungs, liver, kidneys, and spleen. Usage of echocardiography for the same patient revealed fungal balls and vegetation. Both children had pre-B acute lymphoblastic leukemia and were undergoing treatment in the induction phase (ALL). Both patients improved after a combination of antifungal treatments. One child had disseminated *Candida albicans* isolated from blood and ascetic fluid during the induction phase. Despite receiving combined therapy, she died. Another child with *A. flavus* and *A. fumigatus* co-infection developed hand osteomyelitis, which was treated after examining a biopsy that revealed fungal hyphae. Nevertheless, she died too, despite being on combined therapy.

Positive fungal cultures were found in the BAL of three patients, 37.5% of whom had positive blood cultures while 3% of them had positive urine cultures for neonatal invasive candidiasis. One patient had a positive *Candida albicans* urine culture but was not on a Foley catheter and complained of oliguria. In three patients, invasive aspergillosis caused pulmonary IFD, accounting for 9.3% of all infection sites. Candidaemia was found in 28% of IFD patients, and the most common organism was *Candida tropicalis* (15.6%), followed by Candida parapsilosis (6.25%). 

### Treatment and outcome

In addition to antibiotics for febrile neutropenia, which include antipseudomonal coverage with/without aminoglycosides and vancomycin for patients with central lines, antifungal therapy should be initiated if the patient remains febrile with no clear focus beyond 96 h of admission, along with a fungal work up for leukemic patients. The reason is that delays are always associated with increased mortality due to invasive mold infections. 

In the present study, 43% of patients began antifungal therapy during the induction phase, 15% during consolidation and maintenance, 19% during intensification for relapsed patients, and 6% during the fludarabine, cytarabine, granulocyte-colony stimulating factor, and idarubicin (FLAG-IDA) protocol for relapsed AML. Before the collection of the samples, antifungal therapy was mostly used, resulting in negative BAL cultures. In 31% of cases, including those with complicated fungal infections, patients were given a combination of antifungal medications, while monotherapy was provided for 69% of cases. The average antifungal duration was 43 days, with a median of 61 days. Voriconazole was provided for all patients for a median of 42 days. Liposomal amphotericin B was used in the treatment of our patients (66%) with a median treatment time of 11 days (Table 2).

**Table 2:** Results from episodes of candidemia

Days	Agents				
	Voriconazole	Liposomal Amphotericin B	Caspofungin	Fluconazole	Posaconazole
Mean	49.5	18.72	6.22	0.34	1.34
Median	41.5	10.5	0	0	0
Minimum	4	0	0	0	0
Maximum	210	97	67	11	43

## Discussion

In this study, a large sample of IFI data in leukemia children was examined, providing a useful evaluation of IFI epidemiology and outcomes in Oman. To the best of our knowledge, this was the first study that investigated the spectrum, frequency, associated factors, and outcome of IFIs in critically ill children with leukemia in Oman. In the current study, the overall incidence rate of all IFIs in pediatric patients at the Royal Hospital was 16.1% (32/198), including ALL, AML, mixed phenotype, relapsed AML, infantile ALL, and relapsed ALL. These cases were either proven or probable, according to EORTC/MSG guidelines [ 15 ]. Acute Lymphoid leukemia was significantly more common among children (68.75%, 22/32), followed by AML (12.5%, 4/32), infantile ALL (6.25%, 2/32), and relapsed ALL (6.25%, 2/32), and one case of biphenotypic and relapsed AML 3.125% (1/32). Data on the incidence, prevalence, epidemiology, diagnosis, treatment, and outcome of IFI in children with hematological malignancies or undergoing allogeneic HSCT are important since they can help with antifungal management.

Previous studies have looked at the prevalence of IFI in pediatric leukemia patients as well as the incidence of hematologic malignancies all over the world [ 14 - 23 ]. In pediatric patients, the incidence of invasive fungal infection has been reported to range between 1.7% and 35.4%, and the incidence of invasive fungal infection in the present study falls within this range [ 14 - 23 ]. Differences in study populations, hospital conditions, use of prophylactic antifungal agents, and IFI definition criteria may explain the disparity between studies. Direct comparisons between studies are difficult due to differences in study designs, definitions of IFI, patient populations, and treatment protocols. 

Kobayashi et al. found IFIs in 21.6% (11/51) of cases between 1997 and 2006 [ 21 ]. Rosen et al. reported a prevalence rate of 8.5% of all IFIs between 1991 and 2001[ 3 ], while Mor et al. reported 39.4% (26/66) and 13.6% (9/66) incidence rates of all proven/probable IFIs, respectively [ 1 ]. Moreover, Lehrnbecher et al. reported a 4.9% prevalence of proven/probable IFIs between 1991 and 2001 [ 4 ]. Lin et al. found 20.5% (16/78) IFIs [ 22 ], while Supatharawanich et al. found 10.7% overall IFD prevalence, with ALL, AML, relapsed leukemia, and severe aplastic anemia patients accounting for 8%, 11.4%, 19.3%, and 16%, respectively [ 23 ]. Monsereenusorn et al. recently reported that the incidence rate was 13.9% in one of the tertiary hospitals in Thailand [ 24 ]. There were no major differences between males and females. 

High-risk or relapsed ALL, AML, allogeneic HSCT, chemotherapy intensity, associated comorbidities, neutropenia duration, and length of hospital stay are all risk factors for IFD [ 25 , 26 ]. In addition, children undergoing HSCT for severe aplastic anemia or Fanconi anemia were also at a higher risk of IFD [ 27 ]. Majority of the patients had a central venous catheter (CVC) in place at the time of IFD. The CVC is routinely inserted in all newly diagnosed leukemia patients. However, some studies have shown that the presence of CVC is a risk factor for IFD. Nevertheless, a definitive conclusion cannot be drawn from the present study, and additional comparative studies are needed to prove or disprove this as a risk factor.

Majority of our patients arrived at the hospital with febrile neutropenia, and the remainder developed neutropenia. The initial diagnosis in 20 patients (62.5%) was febrile neutropenia. Moreover, six (18.75%) of the admitted patients had pneumonia, while six (18.75%) had septic shock. However, all patients eventually developed febrile neutropenia. Neutropenia is frequently reported in patients with hematologic cancers [ 24 ]. However, cancer types were not linked to IFD in the present study which only included hematologic malignancies (100%) in children with fever and neutropenia, and the vast majority of the patients in this study developed thrombocytopenia. 

Fungal cultures were positive in the present research for peripheral blood (37%), fine needle aspiration/wound (18%), BAL (3%), and urine (3%). Furthermore, a CT scan of the chest revealed evidence of fungal infection in 21 patients (65.6%) before and after antimicrobial treatment. Besides, it was found that 15 (47%) and 17 (53%) patients had proven and probable fungal infections. In 37.5% of IFI patients, a positive blood culture was reported, while 3% had a positive urine culture. Five of the positive fungal blood cultures were *Candida tropicalis*, two were *Candida parapsilosis*, one was *C. albicans*, and one was Candida species. 

One child with positive CT findings for fungal infection had *Aspergillus pleural* fluid cytology. Another child who had positive CT findings and BAL for *A. flavus* also had a necrotic skin lesion that tested positive for the same fungus. The clinical condition of this child deteriorated, and he died in the intensive care unit. Another child with a positive blood culture for *C. tropicalis* had multiple abscesses in the abdomen CT as well as a gluteal abscess. A splenic biopsy revealed a fungal granuloma, and histopathology confirmed the fungal structure. Another child with a positive blood culture for *C. tropicalis* had CT findings of multiple micro-abscesses in the lungs, liver, kidneys, and spleen. In addition, echocardiography showed fungal balls and vegetation. Both children were in the induction phase and had pre-B ALL. Both patients improved after receiving combination therapy. The child who had a positive blood culture for *C. albicans* also had a positive ascetic fluid for the same fungus and was in the induction phase. Despite receiving combination therapy, she died in the PICU. 

Another child with blood cultures positive for both *A. flavus* and *A. fumigatus* developed hand osteomyelitis, had a biopsy, and was found to have fungal hyphae by histopathology. This child had CT findings that suggested a fungal infection, had common ALL, and was in the consolidation phase. Despite receiving combination therapy, she died in the PICU. Finally, one patient had a positive urine culture for *C. albicans* but was not on a Foley catheter and complained of oliguria. 

In this study, 15 patients had proven fungal infection (47%), and 17 patients had probable fungal infection (53%). During the study period, two patients had two episodes of invasive fungal infections, while others had one. Celkan et al. in a multicentric study performed in Turkey in 2019 reported that 8 out of 40 (22.5%) children with HSC had *Candida* spp., with only one case being C. albicans [ 28 ]. Another Turkish study recently reported that *C. parapsilosis* is the most common pathogen in leukemic children [ 29 ]. The most commonly isolated pathogens in the current study were also non-albicans, namely *C. tropicalis* (n=5) and *C. parapsilosis* (n=2), while only one patient had *C. albicans*. 

*Candida albicans*, which once was the most reported pathogen, appears to have been replaced by *C. parapsilosis*, *C. tropicalis*, and other non-albicans species. These findings are consistent with those of recent reports that show a decrease in *C. albicans* infections and an increase in non-albicans infections over the last decade [ 1 , 18 , 29 - 33 ]. *Candida tropicalis*, *C. parapsilosis*, *C. albicans*, other Candida species, *A. flavus*, and *A. fumigatus* were the causative agents of fungal infections in the present study. When treating children with suspected IFD, it is critical to begin empiric antifungal therapy as soon as possible. Preventive treatment is the standard strategy used in our institution for avoiding antifungal overuse [ 28 , 32 , 34 ]. Although echinocandins are the first-line agents for candidemia in children and adults, liposomal amphotericin B and voriconazole may be used if more mold coverage is required [ 35 ]. 

Voriconazole and liposomal amphotericin B should be used to treat fungal infections in leukemia and hematopoietic patients, according to the European Conference on Infections in Leukaemia (ECIL-6) guidelines for treatment of invasive candidiasis, aspergillosis, and mucormycosis [ 36 ]. Voriconazole was used in the present research to treat febrile neutropenia after 4 days of antibiotics. Voriconazole was administered to all patients, with a median duration of use of 42 days. Liposomal amphotericin B was provided for 21 patients (66%) for a median of 11 days. Caspofungin was administered to eight patients, and posaconazole and fluconazole were administered to one patient each. Approximately 58% of the IFD patients in this study required ICU admission (Table 1), and the fatality rate in this cohort was 16%, which is less than what has been reported in other studies [ 28 , 34 , 37 ]. Since some of our patients required a prolonged course of antifungal therapy and others required combined antifungal treatment, a combination of antifungals was used to treat 31% of patients as first or second-line therapy. Voriconazole and liposomal amphotericin B were the most used antifungal therapies in the patients in the present study.

This study had some limitations, including the fact that the data was collected retrospectively and there was incomplete documentation of patient details in their records. Host factors, clinical criteria, imaging techniques, and laboratory procedures were used to classify the patients. The second limitation was that patients were not being followed up on for a variety of reasons. The third limiting factor was that some parents refused certain invasive diagnostic procedures, such as bronchoscopy or biopsy, which could have an impact on diagnosis and treatment. Finally, the incidence rate of IFI in this review may not accurately report the true incidence due to underdiagnosis and missed IFI during the study period. 

## Conclusion

This study provided a clear picture of IFD in children with leukemia, as well as an informative assessment of IFI epidemiology and outcomes in Oman. The findings could help researchers better understand IFD in these patients. It should be mentioned that fungal infections increased during the study period. Comprehension of IFD in the context of local epidemiology is critical for the implementation of appropriate therapeutic interventions early on and the improvement of the survival rate of these children.
